# Design of Apoptotic Cell-Inspired Particles as a Blood Coagulation Test

**DOI:** 10.3390/biomimetics9060367

**Published:** 2024-06-17

**Authors:** Liang Yue, Yasuhiro Nakagawa, Mitsuhiro Ebara

**Affiliations:** 1 Research Center for Macromolecules and Biomaterials, National Institute for Materials Science (NIMS), Tsukuba 305-0044, Ibaraki, Japan; 2 Graduate School of Pure and Applied Sciences, University of Tsukuba, Tsukuba 305-8577, Ibaraki, Japan; 3 Graduate School of Advanced Engineering, Tokyo University of Science, Katsushika, Tokyo 125-8585, Japan

**Keywords:** blood coagulation, biomimetic, apoptotic cells, APTT, MPS

## Abstract

The blood coagulation test is an indispensable test for monitoring the blood coagulation and fibrinolysis functions. Currently, activated partial thromboplastin time (APTT) is the most widely used approach to coagulation testing. However, APTT reagents need to be optimized due to the fact that they are unstable, highly variable, and cannot be easily controlled. In this study, we created apoptotic cell-inspired methacryloyloxyethyl phosphorylserine (MPS) particles for blood coagulation as an alternative to conventional APTT reagents. Particle size could be controlled by changing the concentration of the polymer. The blood coagulation ability of particles was stable at different environmental temperatures. Moreover, the procoagulant activity could be enhanced by increasing the concentration to 0.06 mg/mL and reducing the size of the particles to around 900 nm. Fibrin clotted by particles showed no significant difference from that formed by APTT regent Actin FSL. We propose that MPS particles are a potential alternative to Actin FS for the application of blood coagulation tests.

## 1. Introduction

Blood coagulation is a dynamic process of changing blood from a liquid state to a non-flowing gel state, which is an important part of physiological hemostasis. The essence of blood coagulation is the process by which soluble fibrinogen in blood plasma becomes insoluble fibrin [1,2]. The blood coagulation cascade is a sequence of physiological reactions that culminate in the formation of thrombin. This process involves multiple steps, many of which require the presence of non-enzymatic cofactors [3]. These cofactors are essential for the cascade’s proper function and are categorized into two main classes: clotting factors and negatively charged surfaces [4]. Phosphatidylserine (PS) exposure on platelet membranes is central to the regulation of blood coagulation and thrombin production [5]. It is widely accepted that the negatively charged surface of PS-containing platelet-derived membranes contributes significantly to this rate enhancement. However, the precise mechanism through which this occurs is still debated [6].

Recent advances in viscoelastic assays, such as thromboelastography and rotational thromboelastometry, provide a more comprehensive assessment of coagulation by continuously monitoring clot formation and breakdown. However, conventional screening tests like prothrombin time (PT) and activated partial thromboplastin time (APTT) remain widely used as the standard laboratory tests for evaluating coagulation defects [7,8]. While PT and APTT are automated and convenient, they have significant limitations as static in vitro assays that use crude activators and cannot replicate the intricate processes of coagulation activation and propagation in vivo [9]. Specifically, the APTT test utilizes a variety of commercial reagents consisting of different contact activators, phospholipid sources, and concentrations, leading to substantial variability in results [10,11,12,13,14]. For instance, the use of different platelet-derived phospholipids results in poor consistency of APTT clotting times, which hampers clinical interpretation and decision making on the bleeding risks of patients. Moreover, the clotting times derived from these reagents show poor correlation with thrombin generation profiles in patient plasma. Therefore, the APTT assay may fail to identify some coagulation abnormalities. To address the limitations of existing reagents and more accurately identify coagulopathies, it is imperative to develop novel APTT reagents with standardized phospholipid compositions and activators that can closely mimic physiological coagulation. In this study, we aim to formulate and validate new APTT reagents with optimized phospholipids and activators to minimize assay variability and establish reference ranges that better guide clinical diagnosis and management of coagulation disorders.

To overcome the limitations of conventional APTT reagents, the purpose of this study was to investigate the procoagulant effect of apoptotic cell-inspired polymeric particles as a biomimetic material to affect the blood coagulation system. In our previous study, apoptotic cell membrane-inspired methacryloyloxyethyl phosphorylserine (MPS) particles were helpful for immunosuppressive and post inflammatory effects on macrophages [15]. Phosphatidylserine (PS) is known for its anti-inflammatory effects as well as its role in blood coagulation. In our previous research, we demonstrated that synthetic MPS particles exhibit similar anti-inflammatory effects as natural PS, effectively modulating immune responses in macrophages. Due to these physiological functions, it is expected that these MPS particles will also influence the blood coagulation process. Herein, biomimetic MPS particles are proposed in this study to approach the commercial APTT reagent for comparing procoagulant activity.

## 2. Materials and Methods

### 2.1. Materials

Butyl methacrylate (BMA), hydroxyethyl methacrylate (HEMA), 2,2′-azobis(isobutyronitrile) (AIBN), and imidazole hydrochloride were purchased from Wako Pure Chemical Industries, Ltd. (Osaka, Japan). *O*-tert-butoxy-*N*,*N*,*N*,*N*,-tetraisopropyl phosphoramidite, tert-butyl peroxide, aluminum chloride hexahydrate, calcium chloride, ellagic acid, and glutaraldehyde solution, 25% in H_2_O, were purchased from Sigma-Aldrich (St. Louis, MO, USA). Trifluoroacetic acid (TFA) and chloroform-d were purchased from Tokyo Chemical Industry (Tokyo, Japan). *N*-Boc-l-serine *tert*-butyl ester was purchased from Watanabe Chemical Industries (Hiroshima, Japan). Phosphate-buffered saline and sodium hydroxide were purchased from Nacalai tesque (Kyoto, Japan). Coagtrol N and Actin FSL kits were purchased from Sysmex Corporation (Kobe, Japan). *N*-2-hydeoxyethylpiperazine-*N*′-2-ethane sulfonic acid (HEPES) buffer solution was purchased from Thermo Fisher Scientific (Waltham, MA, USA). Other reagents and solvents are commercial extra-pure grade and were used without further purification.

### 2.2. Synthesis of Poly (BMA-co-HEMA-co-MPS)

**Scheme 1:**
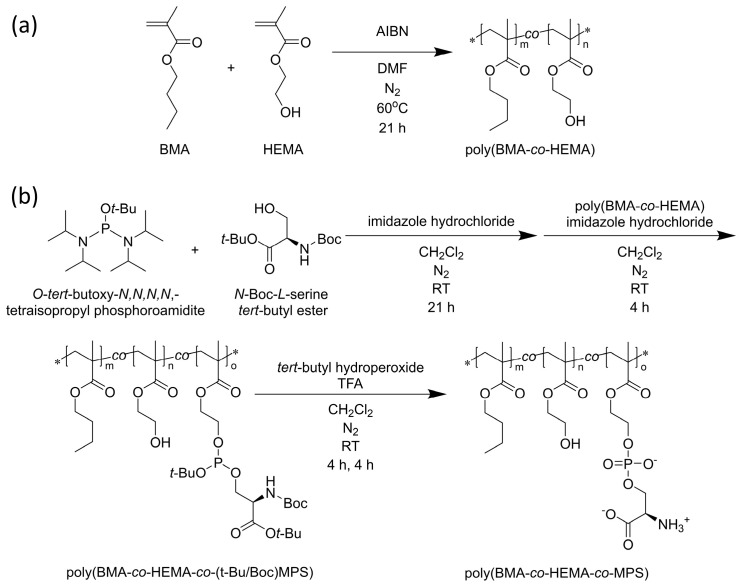
(**a**) Synthesis of poly (BMA-co-HEMA) and (**b**) poly (BMA-*co*-HEMA-*co*-MPS). * indicates the abbreviation of terminal group.

A random copolymer of BMA, HEMA, and MPS was synthesized by previous methods [14,15,16]. Briefly, poly (BMA-*co*-HEMA) was polymerized by free radical polymerization with 7.5 mmol of BMA, 35 mmol of HEMA, and 0.04 mmol of AIBN in 40 mL of DMF at 60 °C for 21 h (Scheme 1a).

Subsequently, the hydroxyl group of serine, whose amine and carboxyl groups are protected, was modified to the side chain hydroxyl group of the resulting poly (BMA-*co*-HEMA) using a phosphoramidite reagent, a phosphate ester-forming reagent. Specifically, 9 mmol of *O*-tert-butoxy-*N*,*N*,*N*,*N*,-tetraisopropyl phosphoramidite, 10 mmol of *N*-Boc-l-serine *tert*-butyl ester, and 2.2 mmol of imidazole hydrochloride were stirred in 200 mL of super-dehydrated dichloromethane in a N_2_ atmosphere for 21 h at room temperature. poly (BMA-*co*-HEMA) was placed in the system, and a total of 28 mmol of imidazole hydrochloride was added in three equal portions that were spaced out by 45 min each under N_2_ atmosphere. At 150 min after the last addition of imidazole hydrochloride, the mixture was purified by dialysis against 2-propanol four times and against dichloromethane twice at 4 °C using a cellulose dialysis membrane (MWCO = 1000). A small part of the solution was measured by ^1^H-NMR spectroscopy to evaluate the modification degree of the PS group.

To the resulting mixture, 22 mmol of *tert*-butyl peroxide was added. The mixture was stirred at room temperature for 6 h, followed by purification through dialysis against dichloromethane twice at 4 °C (MWCO = 1000). Next, trifluoroacetic acid was added to the solution, which was then stirred at room temperature for another 6 h. The mixture was further purified by dialysis against a 0.1 M NaOH solution twice, followed by isopropanol twice, and methanol twice, all at 4 °C (MWCO = 1000). Finally, poly(BMA-*co*-HEMA-*co*-MPS) was obtained by removing the solvent using a rotary evaporator and a vacuum pump overnight (Scheme 1b).

The modification of the PS group was confirmed by measuring the FT-IR spectrum to detect the stretching vibration of PO^2−^ in the polymer. 

^1^H-NMR of poly (BMA-*co*-HEMA): (CDCl_3_-d, 300 MHz): δ = 0.80–1.10 (-CH_3_, -CH_3_ in the chain, broad, (6_m_ + 3_n_)H), δ = 1.40 (-CH_2_-CH_3_, s, 2_n_H), δ = 1.60 (-CH_2_-CH_2_- CH_3_, s, 2_m_H), δ = 1.80–2.00 (-CH^2^-C(CH^3^)(COO-)CH^2^- in the chain, broad, 2_(m + n)_H), δ = 3.80 (-CH_2_-OH, s, 2_n_H), δ = 4.00 (-COO-CH_2_- in the BMA site, s, 2_m_H), δ = 4.10 (-COO-CH_2_- in the HEMA site, s, 2_n_H). GPC: Mn = 12.9 × 10^3^ g mol^−1^, Mw/Mn = 1.13.

^1^H-NMR of poly (BMA-*co*-HEMA-*co*-MPS) before deprotection: δ = 0.80–1.10 (-CH_3_^,^ -CH_3_ in the chain, broad, (6_m_ + 3_n_ + 3_o_)H), δ = 1.30–1.70 (-CH_2_-CH_3_, s, (2_n_ + 2_o_)H; -CH_2_-CH_2_-CH_3_, s, 2_m_H; *tert*-Bu group, multi, 27_o_H), δ = 1.80–2.40 (-CH^2^-C(CH^3^)(COO-)CH^2^- in the chain, broad, 2_(m + n + o)_H), δ = 3.80–4.10 (-CH_2_-OH, s, 2_n_H); (-COO-CH_2_- in the BMA site, s, 2_m_H), δ = (-COO-CH_2_- in the HEMA and PMS site, s, 2_(n + o)_H). GPC: Mn = 8.30 × 10^3^ g mol^−1^, Mw/Mn = 1.18.

### 2.3. Preparation of PS Particles of Poly (BMA-co-HEMA-co-MPS)

The PS particle was prepared through a self-assembly mechanism adopting simple dialysis following the previous method with small modifications [7,9]. Poly (BMA-*co*-HEMA-*co*-MPS) was dissolved in three different concentrations at 2 mg/mL, 4 mg/mL, and 8 mg/mL in DMF, and solutions were dialyzed against distilled water for 3 days at 4 °C using a dialysis membrane (MWCO = 1000). The water was replaced every 12 h. Finally, the sample solution in the dialysis membrane was collected into a glass vial and freeze dried for 3 days to achieve a white powder.

### 2.4. Morphology, Size, and Zeta Potential of PS Particles

Sonification was applied to the three different diluted PS particles in DI water (1 mg/mL) in a water bath desktop ultrasonic cleaner US100 series (SANSYO Co., Ltd., Company, Tokyo, Japan) for about 30 min. Before the operation, the measurement duration was set to be determined automatically. The morphology, size, and zeta potential of PS particles were evaluated by field emission-scanning electron microscopy (FESEM; Hitachi S-4700 I, Tokyo, Japan) and a zeta potential laser/particle analyzer (Dynamic light scattering (DLS), Otsuka Electronics Co., Ltd., Osaka, Japan), respectively (Figures 3 and 4). 

The SEM samples were prepared by being mounted on freeze-dried samples to aluminum SEM pins and coated with Au/Pd using a sputter coating instrument provided at 20 mA for 120 s (Figure 6).

The zeta potentials of PS particles (prepared at a concentration of 2 mg/mL) were evaluated at different concentrations of 0.0006 to 0.6 mg/mL by following the above procedure (Figure 7). 

All of the measurements were performed at room temperature. The software provided by the manufacturer was used to automatically calculate the size of the particles and the polydispersity index. The diameter mean values were calculated from the measurements performed at least in triplicate.

### 2.5. Evaluation of Procoagulant Efficiency of PS Particles by APTT Assay

The influence of particle size and concentration on blood coagulation was evaluated using the Activated Partial Thromboplastin Time (APTT) assay. Coagtrol N was prepared by dissolving it in 1 mL of deionized (DI) water and gently overturning for 30 min at room temperature to ensure stable reagents and remove the bubbles. Ellagic acid was dissolved in a 50 mM HEPES buffer solution (pH 7.35) to a final concentration of 0.1 mM and mixed with 50 μM aluminum chloride hexahydrate as an antioxidant.

To assess the size-dependent effects on coagulation, PS particles of three different sizes (907 nm, 937 nm, and 976 nm) at a fixed concentration of 0.06 mg/mL were tested (Figure 4). Additionally, the influence of particle concentration was explored using PS particle solutions at varying concentrations (0.0006, 0.006, 0.06, 0.6, 2, 4, and 8 mg/mL) (Figure 5). For both sets of experiments, 50 μL of each PS particle solution or concentration variant was mixed with 50 μL of pre-treated Coagtrol N in a UV–visible cuvette. Each mixture was incubated at 37 °C in a water bath for 2 min, followed by the addition of 25 mM CaCl_2_ (50 μL). The clotting time for each sample was then determined. 

The coagulation tests using Actin-FSL and PS particles were conducted throughout the year, specifically in February, May, July, and October of 2020. This approach aimed to demonstrate the fact that PS particles produce stable results across all seasons and environments (Figure 7).

For the blank control group, the procedure was slightly modified: 50 μL of Coagtrol N was pre-warmed in a UV–visible cuvette at 37 °C for 1 min before adding 50 μL of the ellagic acid-based HEPES buffer solution. After a further incubation of 2 min at 37 °C, CaCl_2_ (25 mM, 50 μL) was added, and the clotting time was calculated based on the change in absorbance at 500 nm. The coagulation time was defined from the moment CaCl_2_ was added until the absorbance reached half of the maximum difference observed after reagent addition. The normal reference ranges for the APTT were confirmed according to the Actin FSL reagent kit instructions, serving as a positive control.

All statistical analyses were carried out using Tukey methods.

### 2.6. Preparation of Blood Clots Specimen

To investigate the morphological differences in the fibrin network and fiber structure following the addition of Actin FSL and PS particle solutions, scanning electron microscopy (SEM) was employed for high-resolution visualization. For both the Actin FSL and PS particle groups, fibrin clots were carefully transferred from the UV–visible cuvette to polystyrene cell culture dishes for fixation. The fixation process involved immersing the specimens in phosphate-buffered saline (PBS) containing 2% glutaraldehyde for 60 min at room temperature. Following fixation, the samples were washed briefly in PBS twice for 5 min each, and then rinsed in distilled water twice for 5 min each. The specimens underwent a series of dehydration steps in ethanol solutions of increasing concentrations (70%, 95%, and 100%), spending 10 min in each solution, followed by a 10-min immersion in pure acetone. After dehydration, the specimens were air-dried for 60 min at room temperature. The dried specimens were then mounted on aluminum stubs and sputter-coated with gold/palladium for 1 min (SC-701 MKII, Tokyo, Japan) to prepare them for SEM observation. The acceleration voltage was 20 kV (Figure 3d–f).

## 3. Results

### 3.1. Synthesis and Characterization of Polymers

**Figure 1:**
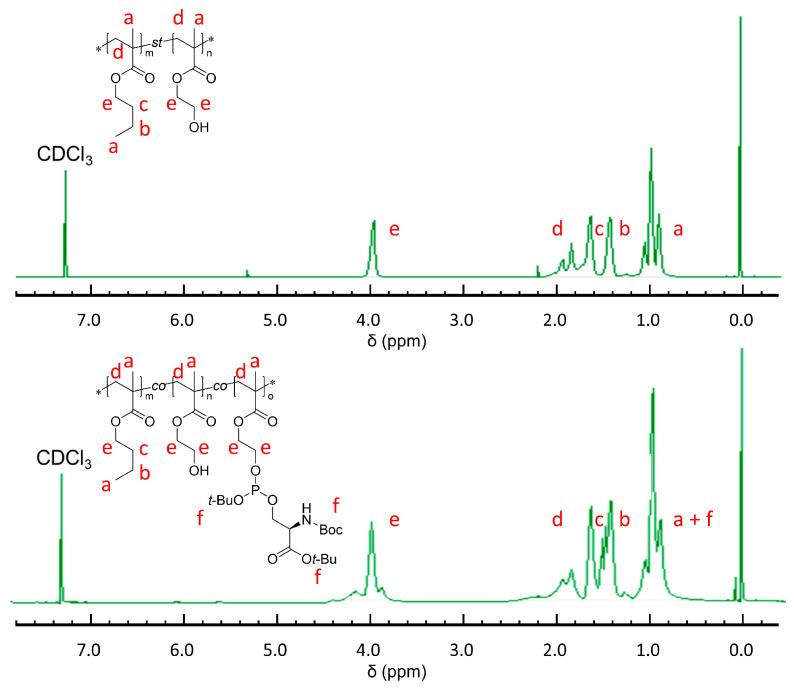
^1^H-NMR spectra of poly (BMA-*co*-HEMA) (**above**) and poly (BMA-*co*-HEMA-co-MPS) (**below**). * indicates the abbreviation of terminal group.

Given the efficiency and hydrophobicity of BMA, alongside the biocompatibility and easy polymerization characteristics of HEMA, we selected these monomers to synthesize the poly (BMA-*co*-HEMA) copolymer through free radical polymerization as precursor materials for PS particles. This approach to modifying polymer materials with PS groups illustrates a foundational principle for various applications, given their recognized biocompatibility. We safeguarded the amine and acid functional groups of the serine using BOC and tert-butyl protective groups, which can be easily removed under mild acidic conditions without affecting side chains. Furthermore, by adjusting the monomer reaction ratio, we could finely tune the hydrophobicity of the polymers, leveraging the phosphoramidite method. For this research, we conducted the polymerization at a molar ratio of 80 mol% BMA to 20 mol% HEMA, a balance that has been effectively validated in our prior studies. Following the application of the phosphoramidite method to the obtained BMA-HEMA copolymer, the final contents of BMA, HEMA, and MPS were determined to be 76.9, 21.0, and 2.1 mol%, respectively. This composition was confirmed using ^1^H-NMR spectroscopy (Figure 1).

**Figure 2:**
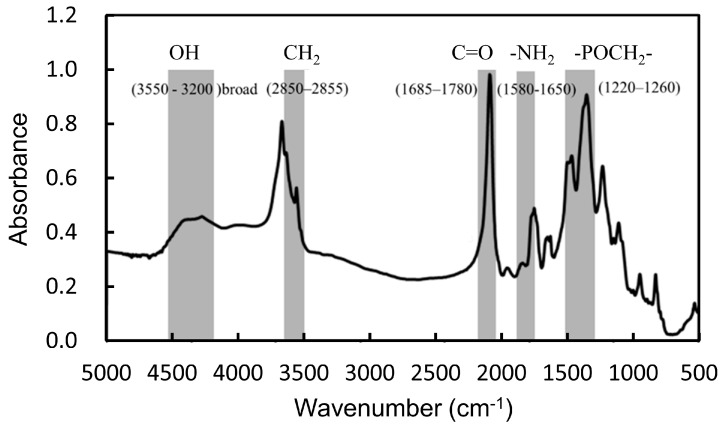
FTIR spectrum of poly (BMA-*co*-HEMA-*co*-MPS).

Poly (BMA-*co*-HEMA-*co*-MPS) was synthesized by a post-polymerization reaction via the phosphoramidite method with deprotection and oxidation of the phosphatidylserine (PS) using *tert*-butyl peroxide and trifluoroacetic acid. The poly (BMA-*co*-HEMA-*co*-MPS) was successfully synthesized as shown in Figure 4, which was investigated using ^1^H-NMR spectroscopy. The existence of phosphate moieties on PS-modified polymers was indicated by FTIR characterization in Figure 2. FTIR spectroscopy is a flexible and highly informative technique extensively used for studying the interactions of bilayer membranes at the water–lipid interface [17]. Each vibration of the phospholipid group has a special frequency, absorption maximum, and width. The FTIR spectrum of poly (BMA-*co*-HEMA-*co*-MPS) was confirmed in the following regions: phosphate asymmetric stretching vibration PO_2_^−^ (1220–1260 cm^−1^), carbonyl ester stretching mode (1685–1780 cm^−1^), the headgroup (carboxylate antisymmetric stretching band at (1640–1620 cm^−1^), and NH_3_^+^ antisymmetric bending at 1620–1600 cm^−1^) [18]. 

### 3.2. Effect of PS Particle Preparation Concentration on Physicochemical Property and Coagulation Time 

**Figure 3:**
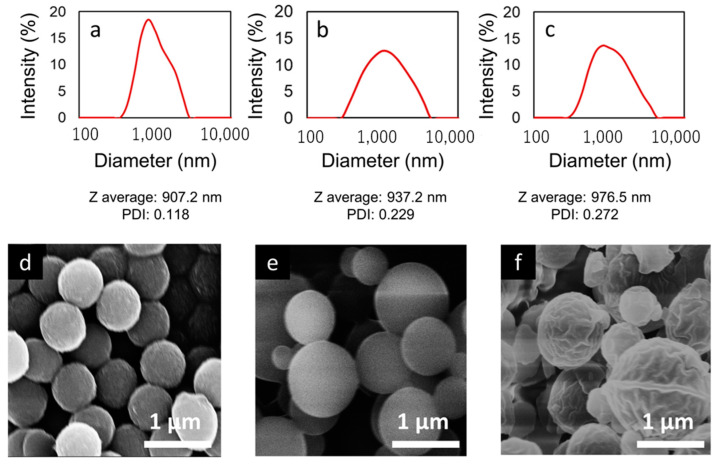
DLS results (**a**–**c**) and SEM images (**d**–**f**) of PS particles preparation with different dialysis conditions (2 mg/mL, 4 mg/mL, and 8 mg/mL in DMF, respectively).

To investigate the effect of polymer concentration on PS particles during preparation, PS particles were prepared in three concentrations (2, 4, and 8 mg/mL in DMF). The average size, size distribution, and polydispersity index (PDI) were measured by the Dynamic Light Scattering (DLS) method in DI water (Figure 3a–c). This analysis was further supported by scanning electron microscopy (SEM) images, presented in Figure 3d–f. 

The size distributions of PS particles prepared at concentrations of 2, 4, and 8 mg/mL are shown in Figure 3a–c. The Z-average diameters of the PS particles were found to be 907.2 nm, 937.2 nm, and 976.5 nm, respectively, with corresponding PDIs of 0.118, 0.229, and 0.272. There was a clear trend where the Z-average particle size decreased with lower polymer concentrations, and similarly, the polydispersity index (PDI) also tended to decrease. This suggests that particles were more monodisperse at lower concentrations, indicating a direct correlation between the concentration of the polymer solution and the uniformity of particle sizes produced during the preparation of PS particles.

Figure 3d–f displays SEM images of PS particles prepared at concentrations of 2, 4, and 8 mg/mL. There is a noticeable trend towards smaller particle sizes at lower concentrations. Notably, PS particles that are adjusted to a concentration of 2 mg/mL exhibit a more uniform size distribution compared to those prepared at higher concentrations. These observations are consistent with and supported by the Dynamic Light Scattering (DLS) results, underscoring the influence of concentration on particle size uniformity.

**Figure 4:**
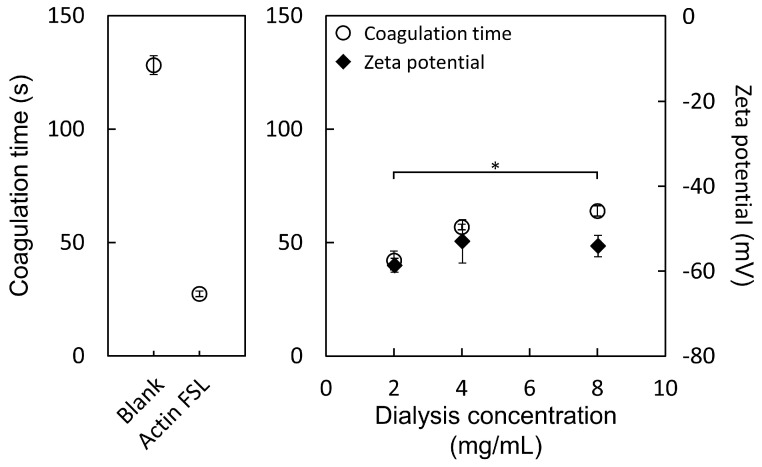
Zeta potential of PS particles prepared at concentrations of 2, 4, and 8 mg/mL in DMF, measured in distilled water at a concentration of 0.6 mg/mL and at room temperature. Measurements were observed at pH 7. * *p* < 0.05.

The zeta potential is recognized as a crucial parameter affecting the stability of nanoparticle systems. Significantly high or low zeta potential values contribute to the stabilization of nanoparticle suspensions. This stabilization occurs because particles carrying similar electric charges repel each other, preventing aggregation and facilitating easier redispersion when necessary. For combined electrostatic and steric stabilization, a zeta potential of at least ±20 mV is preferred, as electrostatic repulsion inhibits the aggregation of particles with identical electric charges. The investigation focused on the zeta potential of PS particles prepared at varying concentrations (2, 4, and 8 mg/mL in DMF) in distilled water at room temperature at a concentration of 0.6 mg/mL (Figure 4). The observed zeta potential ranged between −60 mV and −50 mV at pH 7, with all samples achieving a stable value. These findings indicate a negative surface charge on the PS particles, attributed to the presence of phosphate groups linked to amino acids at the hydroxyl end and the deprotonation of carboxyl and phosphate ester groups on the serine portion near neutral pH levels. Despite no significant differences across the three concentrations, the sample prepared at the lowest concentration (2 mg/mL) exhibited the strongest negative charge in terms of zeta potential. These results underscore the physicochemical stability of the nanoparticle suspension, attributed to the electrostatic repulsion among individual particles.

Various factors related to the formulation and characteristics of nanoparticles significantly impact their application in biological contexts. A critical factor influencing the blood coagulation system is the size of microparticles released from activated platelets. The study illustrated in Figure 4 demonstrates the correlation between the concentration of PS particle preparation and blood coagulation time. It was observed that PS particles exhibit a size-dependent effect on coagulation times, distinct from the blank and Actin FSL groups. Three different sizes of PS particles were evaluated in the APTT (activated partial thromboplastin time) test. The coagulation times were notably reduced by all three sizes of PS particles in comparison to the blank group, indicating a procoagulant activity across all PS particles. Notably, PS particles prepared at the lowest concentration (2 mg/mL), with a size of 906 nm, exhibited superior procoagulant activity compared to those prepared at higher concentrations (4 mg/mL at 937 nm and 8 mg/mL at 977 nm). This finding demonstrates that smaller-sized particles are more effective in promoting blood coagulation than their larger counterparts.

### 3.3. Concentration Dependency of Coagulation Time 

**Figure 5:**
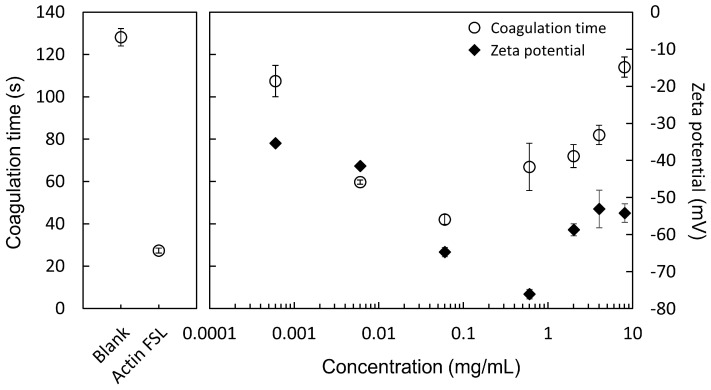
Effects of PS particle concentration on blood coagulation time and zeta potential. PS particles were prepared at 2 mg/mL and tested at various concentrations.

Figure 5 shows that PS particles, prepared at 2 mg/mL, affect blood coagulation time differently and zeta potential based on their concentration. It was found that the PS particles exhibit a concentration-dependent influence on coagulation times when compared to the blank group. Both PS particles and Actin FSL groups were able to significantly reduce the time required for the APTT (activated partial thromboplastin time) test, indicating a similar procoagulant activity between PS particles and Actin FSL. Interestingly, the zeta potential of PS particles was at its minimum when the concentration was 0.6 mg/mL. However, the shortest coagulation time was observed at a concentration of 0.06 mg/mL, suggesting that this specific concentration of PS particles has a more pronounced effect on accelerating the blood coagulation process.

### 3.4. Morphology of Fibrin Clots 

**Figure 6:**
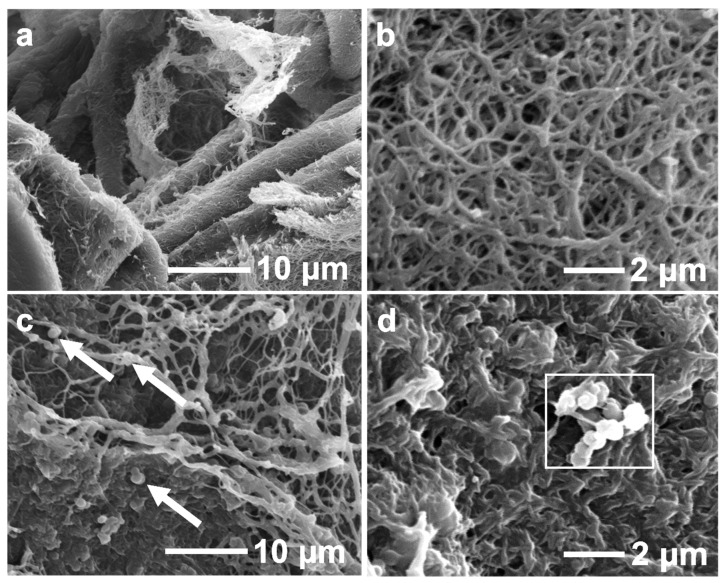
Morphological observation of fibrin clots formation in Actin FSL (**a**,**b**) and 0.06 mg/mL PS particles solution (**c**,**d**) with different magnification. Allows indicate fibrin clots.

The morphological characterization of fibrin clots with Actin FSL and PS particles was analyzed using SEM. Figure 6a,b show fibrin clots from commercial blood plasma (Coagtrol N) at 7000× and 10,000× magnification. Clots with Actin FSL displayed a dense network of thin fibers. Clots with PS particles showed potential PS particles attaching to fibers, as indicated by the arrows in Figure 6c,d. These clots had rough surfaces with sub-micron particles on the fibrins, suggesting phosphatidylserine might bind directly to the fibrins, enhancing the coarse and porous network. PS particles were often near fibril junctions, hinting at their role in fiber-fiber interactions. Fibrin mesh formed around PS particles, indicating fibrin assembly might start near these particles. Fibrin attached to PS particles remained undeformed under experimental conditions. The diameter of the fibrin in Figure 6 was calculated by image analysis. The fibrin diameters prepared using actin FSL and PS particles were 256 ± 28.0 and 250 ± 9.00, respectively. There is no significant difference in the fibrin diameters prepared using Actin FSL and PS particles, suggesting that PS particles can be used as alternatives to Actin FSL. In summary, PS particles promote thrombin and fibrin generation, improve fibrin structure, and integrate into the fibrin network in normal plasma models.

### 3.5. Stability of PS Particles in APTT Test Interpretation

Commercially available APTT reagents display variability in aspects like contact activators, reference values, and phospholipid compositions, leading to challenges in achieving uniform results. The variability in the phospholipid area and fatty acid composition of APTT reagents is significant and difficult to control. It is recommended that new reagents be tested in parallel with existing APTT reagents across multiple runs to ensure consistency. Additionally, the working stability of new reagents under different environmental conditions should be assessed to ensure coagulation time results fall within the acceptable error range for the APTT test.

**Figure 7:**
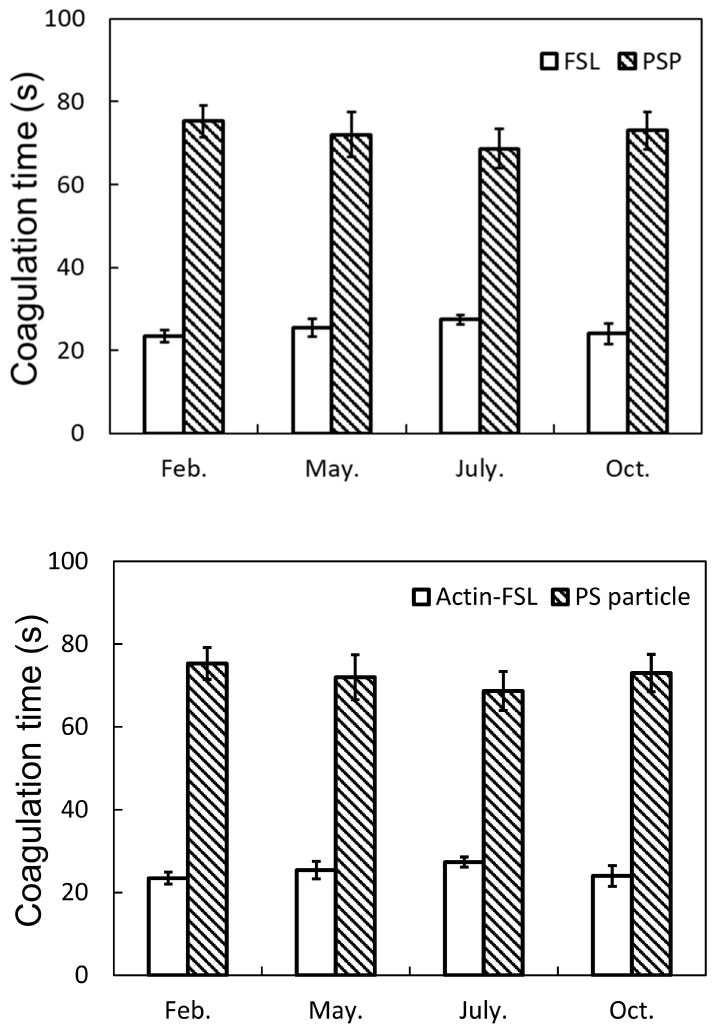
Blood coagulation tests using Actin-FSL and PS particles throughout the year. There were no significant differences in the four timings for each sample (Tukey method).

In this study, APTT tests were conducted under varying environmental conditions using PS particles and Actin FSL. The study focused on the stability of the APTT test for a PS particle solution (2 mg/mL) and a commercial liquid Actin FSL reagent over one year at different environmental temperatures. Figure 7 demonstrated the stability of the APTT test over 1 year, showing no significant difference in coagulation time, indicating the working stability of PS particles. Furthermore, the coagulation time with PS particle solution was significantly shorter than the normal blood clotting time for a healthy human (approximately 3 to 10 min), suggesting that PS particle solution can enhance thrombin generation and improve fibrin formation in the blood coagulation system.

## 4. Discussion

The surface of PS particles prepared at the highest concentration, as seen in SEM images (specifically, Figure 3f), showed wrinkles. This characteristic is considered to be a result of the preparation method rather than an intrinsic property of the particles. The smallest particles, measuring 906 nm in diameter and produced at the lowest concentration of 2 mg/mL, exhibited the most negative zeta potential. Considering that particle size and zeta potential do not have a direct theoretical relationship, a change in surface structure could imply that these particles expose a greater number of PS moieties. A significant difference in coagulation times was observed between particles produced at 8 mg/mL and those at 2 mg/mL, as determined by Tukey’s method. The explanation for this finding is that smaller particles have a larger surface area relative to their volume, likely allowing for more PS moieties to be exposed. Given that PS acts as a trigger in coagulation, this finding is noteworthy. From these observations, it can be concluded that PS particles prepared at a concentration of 2 mg/mL are notably effective. Their smaller size and larger specific surface area mean they expose more PS moieties per unit area than those prepared at higher concentrations. This efficiency in exposing PS moieties suggests they could be more effective in applications where coagulation time is a critical factor.

From the perspective of coagulation time, a concentration of 0.06 mg/mL exhibited the shortest coagulation time. PS groups act as an accelerator in blood coagulation, suggesting a negative correlation between PS particle concentration and coagulation time up to a certain concentration [19]. However, since PS groups strongly trap calcium ions, an essential factor in coagulation, exceeding a certain concentration can deplete calcium ions in the system [20,21]. This depletion would predictably shift the correlation from negative to positive beyond a certain PS particle concentration. In this experiment, the optimal balance was found at a concentration of 0.06 mg/mL. It is important to note that this balance can vary with the particle size and the amount of PS moieties introduced, requiring evaluation with each new batch synthesis.

In the examination of the morphology of fibrin clots, no significant differences were observed between the samples created using Actin-FSL and those with PS particles. The only distinction was the presence of PS particles. In both types of samples, fibers forming a uniform network were identified, suggesting that PS particles may induce coagulation through a mechanism similar to that of coagulation reagents [22,23]. This observation implies that PS particles could be functioning in a manner akin to traditional coagulation factors, facilitating the formation of uniformly structured fibrin clots.

To evaluate the impact of external environmental conditions on the coagulation time of PS particles, the same coagulation test was conducted at four different time points over a year, and no differences were observed. All tests were carried out in Tsukuba City, Japan, in February, May, July, and October. The average temperature, average highest temperature, average lowest temperature, and average humidity for each month in 2020 are summarized in Table 1.

The evaluation conducted at different time points showed no significant difference in coagulation time for Actin-FSL, indicating stable performance across the seasons. Similarly, PS particles exhibited no significant differences at all time points. Although all measurements were conducted in a laboratory where temperature and humidity were controlled to be constant, it was anticipated that there could be slight variations due to the actual seasonal temperatures and humidity levels. Thus, the experiment was not designed to expose the systems to seasonal external environments directly but rather aimed to discuss the potential impact of seasonal variations at the laboratory level on the evaluation. Moreover, the PS particles used in this test were from the same batch and stored in a lyophilized state, indicating that PS particles maintain their activity for at least one year without significant loss of function.

Aizhen Yang et al. successfully demonstrated that apoptotic cells had the ability to accelerate blood coagulation [25]. Aizhen Yang et al. revealed that Factor XII (FXII) preferentially binds to apoptotic cells and is rapidly activated, demonstrating the contribution of apoptotic cells to coagulation enhancement. While thrombin generation is initiated by two pathways associated with vascular damage and blood-derived factors, this research indicates that phosphatidylserine (PS) in apoptotic cells functions as a novel activator of FXII. Furthermore, it was shown that FXII plays a crucial role in coagulation mediated by apoptotic cells. Considering the importance of the rapid clearance of apoptotic cells in maintaining anti-inflammatory and antithrombotic states, especially in the context of increased apoptotic cells due to autoimmune diseases, chemotherapy, and inflammation, this could lead to the development of new therapeutic strategies [26,27]. The findings suggest that PS particles, which mimic apoptotic cells as reported in this study, not only promote blood coagulation through a similar mechanism but also have potential applications in the body since they are cleared from tissues by immune cells with PS receptors, extending beyond coagulants. Such potential of PS can be combined with other therapeutic strategies and pave the way to developing new medicines and medical devices [28,29]. 

## 5. Conclusions

In summary, a novel PS polymer inspired by the apoptotic cell membrane was synthesized successfully. PS group-exposing anti-coagulation particles were obtained by a self-organization procedure. The PS particles showed procoagulant activity effect against normal blood plasma. The procoagulant activity could be enhanced by increasing the concentration to 0.06 mg/mL and reducing the size of PS particles. Such acceleration of normal blood plasma highlights the possibility of the application of PS particles for screening bleeding disorder diseases. 

## Figures and Tables

**Table 1:** Average temperatures and humidity at Tsukuba, Japan in 2020 [24].

	Feb.	May	July	Oct.
Average temperature (°C)	6.3	18.4	23.4	16
Average maximum temperature (°C)	12.4	23.6	26.8	20.6
Average minimum temperature (°C)	0.3	13.7	20.9	12
Average humidity (%)	66	79	92	84

## Data Availability

All data generated or analyzed during this study are included in this article. The raw data are available from the corresponding author upon reasonable request.
